# *QuickStats:* Age-Adjusted Death Rates* for Stroke^†^ Among Adults Aged ≥ 65 Years, by Region^§^ and Metropolitan Status^¶^ — National Vital Statistics System, United States, 2020

**DOI:** 10.15585/mmwr.mm7143a4

**Published:** 2022-10-28

**Figure:**
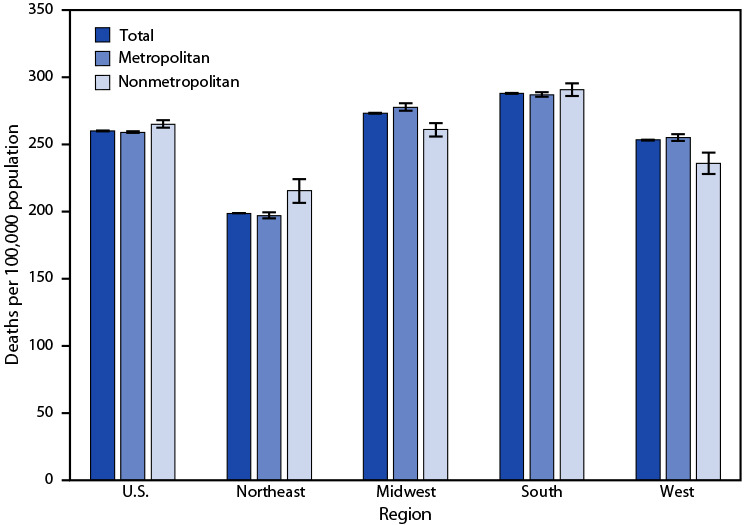
In 2020, the age-adjusted death rate for stroke among adults aged ≥65 years was 260.5 deaths per 100,000 population with rates lower in metropolitan compared with nonmetropolitan areas (259.4 versus 265.5). The rate was highest among those living in the South (288.2) and lowest among those living in the Northeast (199.1). In the Northeast, the death rate for stroke was lower among adults in metropolitan areas (197.4) than in nonmetropolitan areas (215.7). In the Midwest and West, death rates for stroke were higher among adults in metropolitan areas (278.0 and 255.4, respectively) than in nonmetropolitan areas (261.4 and 236.4, respectively). No statistically significant difference was observed between metropolitan and nonmetropolitan areas in the South (287.4 versus 290.9).

**Source:** National Center for Health Statistics, National Vital Statistics System, Mortality Data, 2020. https://www.cdc.gov/nchs/nvss/deaths.htm

